# The value of transhumance for biodiversity conservation: Vulture foraging in relation to livestock movements

**DOI:** 10.1007/s13280-021-01668-x

**Published:** 2021-12-07

**Authors:** Natividad Aguilera-Alcalá, Eneko Arrondo, Roberto Pascual-Rico, Zebensui Morales-Reyes, José M. Gil-Sánchez, José A. Donázar, Marcos Moleón, José A. Sánchez-Zapata

**Affiliations:** 1 Department of Applied Biology, Miguel Hernández University, 03202 Elche, Spain; 2 Centro de Investigación e Innovación Agroalimentaria y Agroambiental (CIAGRO-UMH), Miguel Hernández University, Elche, Spain; 3 Department of Conservation Biology, EBD-CSIC, 41092 Seville, Spain; 4 Research Institute of Hunting Resources (IREC - CSIC, UCLM, JCCM), 13005 Ciudad Real, Spain; 5 Department of Zoology, University of Granada, 18071 Granada, Spain

**Keywords:** Farming intensification, GPS-tracking, Livestock, Traditional farming practices, Vultures, Wild ungulates

## Abstract

In recent decades, intensive techniques of livestock raising have flourished, which has largely replaced traditional farming practices such as transhumance. These changes may have affected scavengers’ behaviour and ecology, as extensive livestock is a key source of carrion. This study evaluates the spatial responses of avian scavengers to the seasonal movements of transhumant herds in south-eastern Spain. We surveyed the abundance of avian scavengers and ungulates, and analysed the factors affecting the space use by 30 GPS-tracked griffon vultures (*Gyps fulvus*). Griffons’ foraging activity increased in the pasturelands occupied by transhumant herds, which implied greater vulture abundance at the landscape level during the livestock season. In contrast, facultative scavengers were more abundant without transhumant livestock herds, and the abundance of wild ungulates did not change in relation to livestock presence. We conclude that fostering transhumance and other traditional farming systems, to the detriment of farming intensification, could favour vulture conservation.

**Supplementary Information:**

The online version contains supplementary material available at 10.1007/s13280-021-01668-x.

## Introduction

Humans and scavengers have maintained a close and changing relationship since our origins (Moleón et al. 2014). With the first hunters and, especially, shepherds, humans became key carrion suppliers to scavenging animals, while scavengers provided humans with an important hygienic service by efficiently removing animal debris (Moleón et al. 2014). In particular, extensive livestock carcasses from traditional exploitations became a staple food source for vultures and other scavengers in many areas (Margalida et al. 2011), and even exceeded the contribution of wild ungulates (Margalida et al. 2018). Thus, traditional extensive livestock farming has helped to sustain scavenger populations in many regions of the world, such as Eurasian griffon vultures (*Gyps fulvus*; Parra and Tellería 2004) and Egyptian vultures (*Neophron percnopterus*; Mateo-Tomás and Olea 2010) in south-western Europe.

However, the profound change in livestock products demand and management in the last few years could strongly influence scavenger behaviour and conservation. Both economic development and human population growth have led to an increasing demand in livestock products and a shift towards industrialization (“livestock revolution”; Seré et al. 2008). This change has involved the stabling of livestock (i.e. intensive farming) and a decline in extensive farming practices, particularly transhumance, which consists of the seasonal movement of herds to take advantage of the availability of natural pastures (Bunce et al. 2004). Transhumant livestock movement takes place twice a year, from wintering to summering areas and back, and is usually based on either latitudinal (north to south in the Northern Hemisphere) or altitudinal travels (generally, from mountains to lowland areas). There is evidence that this farming system has been practised in the Mediterranean region since the Neolithic (6000 years BC; Tejedor-Rodríguez et al. 2021). In Spain, a Mediterranean country with marked historic transhumance activity (MARM 2011), there were 3.5 million transhumant sheep in the eighteenth century, linked with Europe’s wool demand and the Spanish monopoly on merino wool production (Oteros-Rozas et al. 2013a). After the breakdown of this monopoly, transhumance underwent a continuous decline. Today, fewer than 0.5 million of the c. 15.5 million sheep in Spain perform transhumance (MAPA 2019). In parallel, the main market product of transhumant livestock has changed from wool to meat. Among the causes of the current decline of transhumant livestock numbers are the loss of economic profitability compared to intensive systems, rural abandonment and lack of generational relay (Oteros-Rozas et al. 2013a). In order to protect transhumance from disappearing, the Spanish government declared it to be a representative manifestation of intangible cultural heritage (Spanish Royal Decree 385/2017, of 8 April). The United Nations Educational, Scientific and Cultural Organization (UNESCO) also inscribed transhumance on the Representative List of the Intangible Cultural Heritage of Humanity in 2019 (https://ich.unesco.org/en/decisions/14.COM/10.B.2).

Moreover, the abandonment of transhumance and other traditional livestock practices leads to landscape transformation. Lack of livestock grazing may entail vegetation succession, which promotes a landscape change from grassland to shrub and forest by means of a passive rewilding process (Navarro and Pereira 2015; Corlett 2016). Rewilding triggers an effect on the entire ecosystem, from primary producers to tertiary consumers, such as scavengers (Cortés-Avizanda et al. 2015). Shrub and forest landscape has favoured the expansion of wild ungulates in many European countries (Apollonio et al. 2010), such as Spain (Acevedo et al. 2011). However, for vultures and other avian scavengers, finding carcasses in more heterogeneous habitats with greater vegetation cover takes longer than in the open grazing areas occupied by livestock (Arrondo et al. 2019). In addition, intensive livestock farms substantially change the feeding paradigm for vertebrate scavengers by providing more predictable food in both space and time (Moleón et al. 2019). In turn, the food provided by these highly-predictable resources is of low-quality, as it contains veterinary drugs and other pollutants to which scavengers are exposed (Blanco et al. 2019). Thus, vultures inhabiting anthropized areas with intensive livestock farming have poorer physiological conditions (Gangoso et al. 2021) and lower survival rates due to human-cause mortality than those inhabiting more natural areas with more traditional farming systems (Arrondo et al. 2020a). Given vultures’ poor conservation status worldwide (Ogada et al. 2012), investigating the livestock-vulture ecological relationship in the current scenario of rapid global environmental change is an urgent task.

Unlike most avian scavenger species, which are considered facultative or opportunistic scavengers, vultures are obligate scavengers (DeVault et al. 2003), which means that they completely rely on carrion to survive and reproduce. Thus, their foraging and distribution are greatly conditioned by carrion availability (Arrondo et al. 2018). Previous studies based on field surveys provided some evidence that vultures respond to the presence of transhumant livestock, with increased griffon vulture occurrence in roosts close to transhumant herds (Olea and Mateo-Tomás 2009). However, vultures are extremely mobile species (Ruxton and Houston 2004), which challenges in depth investigation of causes and consequences of vulture movement. In recent years, there has been an increase in the use of GPS devices to track large bird movements (Alarcón and Lambertucci 2018). Thus, combining GPS-tracking techniques and field surveys could help to obtain a wide understanding of the ecological processes that shape vulture foraging, at spatial scales ranging from individuals to landscapes.

The present study aims to evaluate the space use by avian scavengers in response to the seasonal movements of transhumant herds in Sierra de Cazorla, Segura y Las Villas Natural Park (Spain), an area that houses the largest number of transhumant sheep in western Europe. To address this, we firstly explored the relationship between the abundance of avian scavengers (both obligate and facultative) and the abundance of wild and domestic ungulates at the landscape level, as inferred by field surveys. We secondly analysed the individual spatial responses of GPS-tracked griffon vultures to the presence and absence of transhumant herds. We focussed on the griffon vulture because it is the most abundant vulture species in Europe (Margalida et al. 2010) and the most efficient consumer of ungulate carcasses in Mediterranean habitats (Mateo-Tomás et al. 2017). Our general hypothesis is that avian scavengers, especially vultures, will arrange their space use according to carrion availability. In particular, we expect that vultures will increase their use of transhumant livestock areas in the season in which transhumant herds are present, while avian facultative scavengers will show a weaker response due to their reliance on alternative food sources.

## Materials and methods

### Study area

This study was carried out in one of Europe’s largest protected areas (the Sierras de Cazorla, Segura y Las Villas Natural Park; 2143 km^2^), a mountain region in south-eastern Spain. The climate is Mediterranean, with annual rainfall ranging between 300 and 1600 mm, and a mean annual temperature of 12–16 ºC. The area is covered mainly by pine (*Pinus halepensis*, *P. nigra*, *P. pinaster* and *P. sylvestris*) and oak (*Quercus ilex* and *Q. faginea*) forests (Rivas-Martínez 1987). The natural park hosts a rich community of vertebrate scavengers. Obligate scavengers include four vulture species: three resident species (griffon, bearded *Gypaetus barbatus* and Egyptian vultures) and one species that is occasionally present (cinereous vultures *Aegypius monachus*) (Morales-Reyes et al. 2017). The griffon vulture is the most abundant vulture in this area, with more than 300 breeding pairs (Del Moral and Molina 2018). Major facultative scavengers include birds like golden eagles (*Aquila chrysaetos*), common ravens (*Corvus corax*), carrion crows (*C. corone*), Eurasian magpies (*Pica pica*) and Eurasian jays (*Garrulus glandarius*), and also mammals like red fox (*Vulpes vulpes*) and wild boar (*Sus scrofa*) (Arrondo et al. 2019). Apart from wild boar, the wild ungulates community includes red deer (*Cervus elaphus*), fallow deer (*Dama dama*), mouflon (*Ovis orientalis*) and Iberian ibex (*Capra pyrenaica*) (Martínez-Martínez 2002).

**Fig. 1:**
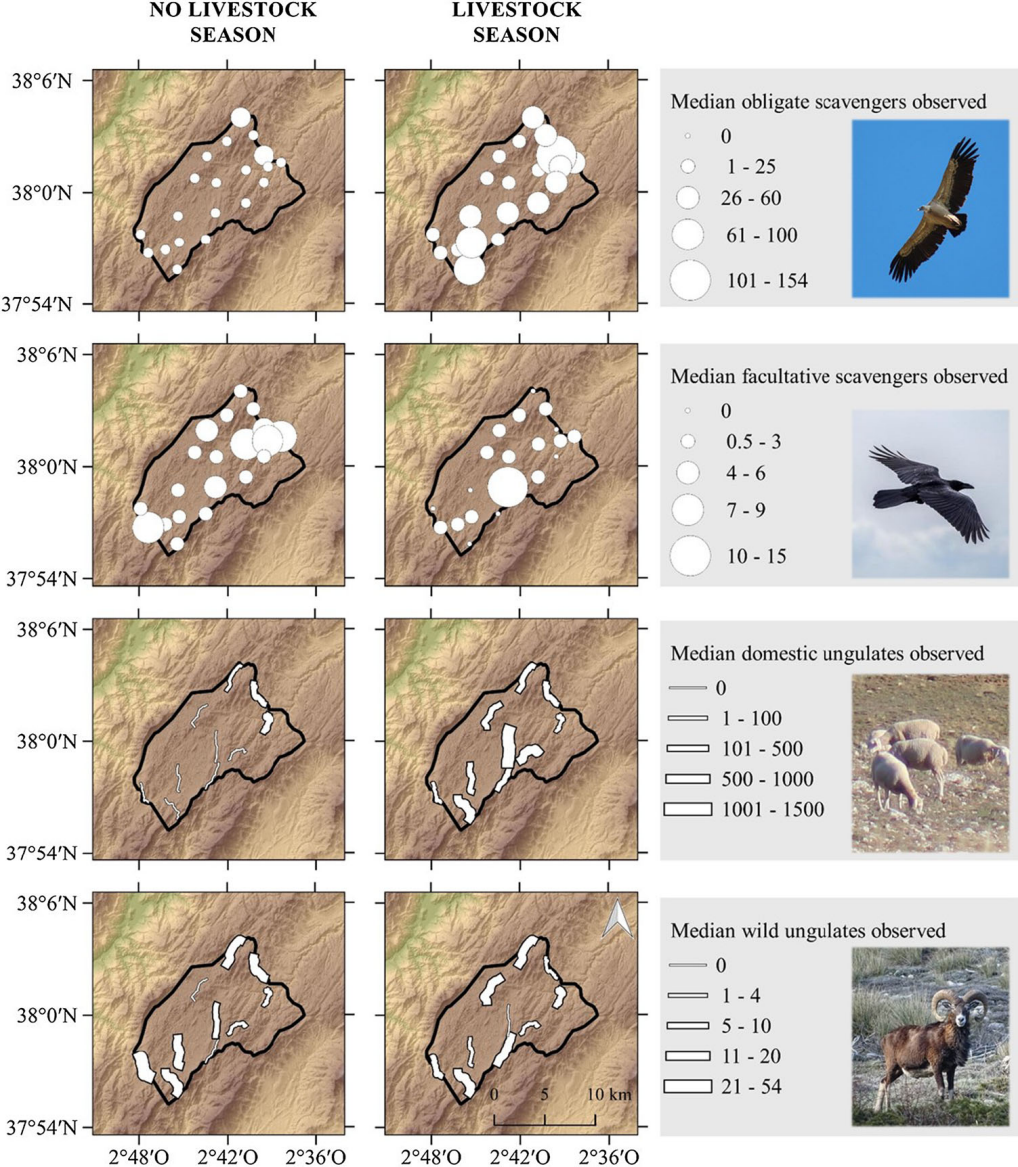
Location of the study area (i.e. the high plateau called “Campos de Hernán Perea”) in the Sierra de Cazorla, Segura y Las Villas Natural Park, south-eastern Spain. Point counts for avian scavengers and transects for ungulates are shown per season (livestock and no livestock). The size of the point counts (circles) and transects (lines) are proportional to the median number of observed individuals

In the highest part of this natural park, there is a plateau called “Campos de Hernán Perea” (150 km^2^; 1600–1700 m a.s.l.; Fig. 1). This plateau is covered by communal pastures where transhumant herds from different lowland ranges in the Sierra Morena mountains (located 50–200 km away) arrive at the end of May and stay until the end of November (Morales-Reyes et al. 2018). This denotes two clearly delimited seasons in relation to the presence (June-November; hereafter “livestock season”) or absence of livestock (December-May; hereafter “no livestock season”). Transhumant herds include c. 35 000 animals (Arrondo et al. 2019) (7% of the total transhumant livestock in Spain; Oteros-Rozas et al. 2013a), mostly (> 90%) sheep (*Ovis aries*), but also goats (*Capra aegagrus hircus*), cattle (*Bos taurus*) and horses (*Equus ferus caballus*).

### Abundance of avian scavengers, livestock and wild ungulates

To determine if transhumance practices affect avian scavengers’ space use, we assessed the differences in the abundance of avian scavengers (both obligate—vultures—and facultative scavengers—other raptors and corvids–) and ungulates (both domestic and wild species) between the seasons with and without transhumant livestock. For birds, we conducted 75 systematic 30-min point counts (for a similar approach, see Moleón et al. 2020). We surveyed 37 points during the livestock season (17 in September–October 2019, 20 in July 2020) and 38 during the no livestock season (18 in January, 20 in February 2020). Point counts were separated by at least 1.2 km from one another (Fig. 1). Surveys were conducted with the help of binoculars (× 12) and field scopes (20–60x) from 2 h after sunrise to 2 pm (UTC + 1) in winter and 3 pm (UTC + 2) in summer to, thus, cover soaring birds’ main active period (Vergara 2010). For each point count, we recorded the observed species and the number of observed individuals. To minimize recounting birds within and among points, we also recorded observation time, distance from the birds to the observer, and their flight direction (Bibby et al. 2000).

In order to quantify seasonal changes in the relative abundance of wild and domestic ungulates, we conducted 40 systematic 3-km-long transects (Putman et al. 2011): 20 during the livestock season (10 in September–October 2019, 10 in July 2020) and 20 during the no livestock season (10 in January, 10 in February 2020). Transects were done by walking on the unpaved roads distributed across the study area (Fig. 1). Transects were carried out at dawn or dusk when ungulates are more active (Pérez et al. 2014). For each transect, we recorded the observed species and number of individuals. To avoid double counting among transects, we also recorded observation time, location of the observed ungulates, and their direction, and their age and sex whenever possible. We expressed the relative abundance of both wild and domestic ungulates according to the Kilometric Abundance Index (KAI), i.e. the number of individuals observed per km walked (Vincent et al. 1991).

Both point counts and transects were distributed throughout the area mostly used by transhumant herds, i.e. the grassland plateau (Fig. 1). The average bird and ungulate abundances were calculated separately for each season (livestock and no livestock).

### Vulture GPS-tracking

Using a baited cannon-net, we captured 30 adult griffon vultures in the natural park in December 2014. Each vulture was equipped with 90 g GPS/GPRS-GSM devices from e-obs digital telemetry (http://www.e-obs.de), which were attached using a backpack harness. These devices were configured according to weather conditions (see Table S1). Birds were monitored between January 2015 and December 2018, unless they died or the device technically failed (Table S2). All the individuals were sexed by molecular procedures (Wink et al. 1998). For each monitored year, the breeding status of all the tracked individuals was determined (breeding vs. non-breeding; see Table S2 for details) and the nest location of all the breeding birds. Breeding status was firstly identified by the accumulation of GPS fixes on cliffs suitable for nesting during vultures’ nestling period (from March to August; Donázar 1993), and was then confirmed by field observations. The number of flying fixes (a proxy for foraging activity) located both inside and outside the plateau used by transhumant herds (see Fig. 1) was identified for this period. Flying fixes were considered those locations with a ground speed > 5 m/s (see Arrondo et al. 2018 for more details).

### Statistical analyses

We firstly used non-parametric Wilcoxon rank tests to explore changes in the abundance of avian scavengers and ungulates between seasons. The absence of normality in these variables was confirmed by the Shapiro–Wilk test (*α* = 0.05).

In order to analyse the influence of transhumant herds on griffon vultures’ use of the study area, we followed two different approaches: one focussed on the landscape level and another on the individual level. In the first approach, the aim was to explore whether griffon vultures were more frequently observed in the livestock area during the livestock season. For this purpose, we used generalized linear mixed models (GLMMs; Zuur et al. 2009) with Poisson error distributions and log link functions. We considered as response variable the “abundance” of griffon vultures observed per point count. The explanatory variables in the fixed term were the livestock “season” (yes/no) and the “nestling” period of griffon vultures (yes/no). To avoid pseudoreplication, we included “point” counts as a random term. We firstly constructed a full model with all the explanatory variables (no interactions among variables were considered due to low sample size). We secondly constructed the set of alternative models with different combinations of the random structure (i.e. one model with a “point” count as a random factor and another with no random term). We used the glmer() function of the *lme4* package in R (Bates et al. 2015). We thirdly selected the model with the most appropriate random structure. Model selection was based on Akaike’s Information Criterion (AIC). After selecting the most appropriate random structure (with a random term in our case), we selected the model with the most appropriate fixed structure by exploring the complete set of alternative models using the dredge() function of the *MuMIn* package in R (Barton 2013). The models with delta-AIC < 2 in relation to the best model (i.e. the model with the lowest AIC) were considered to have similar support (Burnham and Anderson 2002). We finally explored candidate model’s performance by means of marginal *R*^2^, which measures how much variability in the response variable is explained by the model’s fixed term (Nakagawa and Schielzeth 2013). For this purpose, we used the r.squaredGLMM() function of the *MuMIn* package in R (Barton 2013).

In the second approach, which aimed to assess individual griffon vulture foraging activity changes, we explored whether the GPS-tracked griffon vultures used the study area more during the livestock season, and which factors made some griffon vultures to use the area more than others. We applied GLMMs with binomial error distributions and logit link functions. We modelled the response variable as the proportional data with a binomial denominator of the total “GPS fixes” that fell within vs. beyond the study area limits each month per griffon vulture individual. The explanatory variables in the fixed term were livestock “season” (yes/no), “breeding” status (breeding/non-breeding), “sex” (male/female) and “year” (as a factor, one for each tracked year). The individual (“ind.”) was included as a random term. The process for model construction and selection, and for calculating the candidate models’ performance, was like that described above.

All the analyses were done in R software, version 3.6.1 (R Core Team 2019) (https://cran.r-project.org/).

## Results

### Abundance of avian scavengers, livestock and wild ungulates

**Fig. 2:**
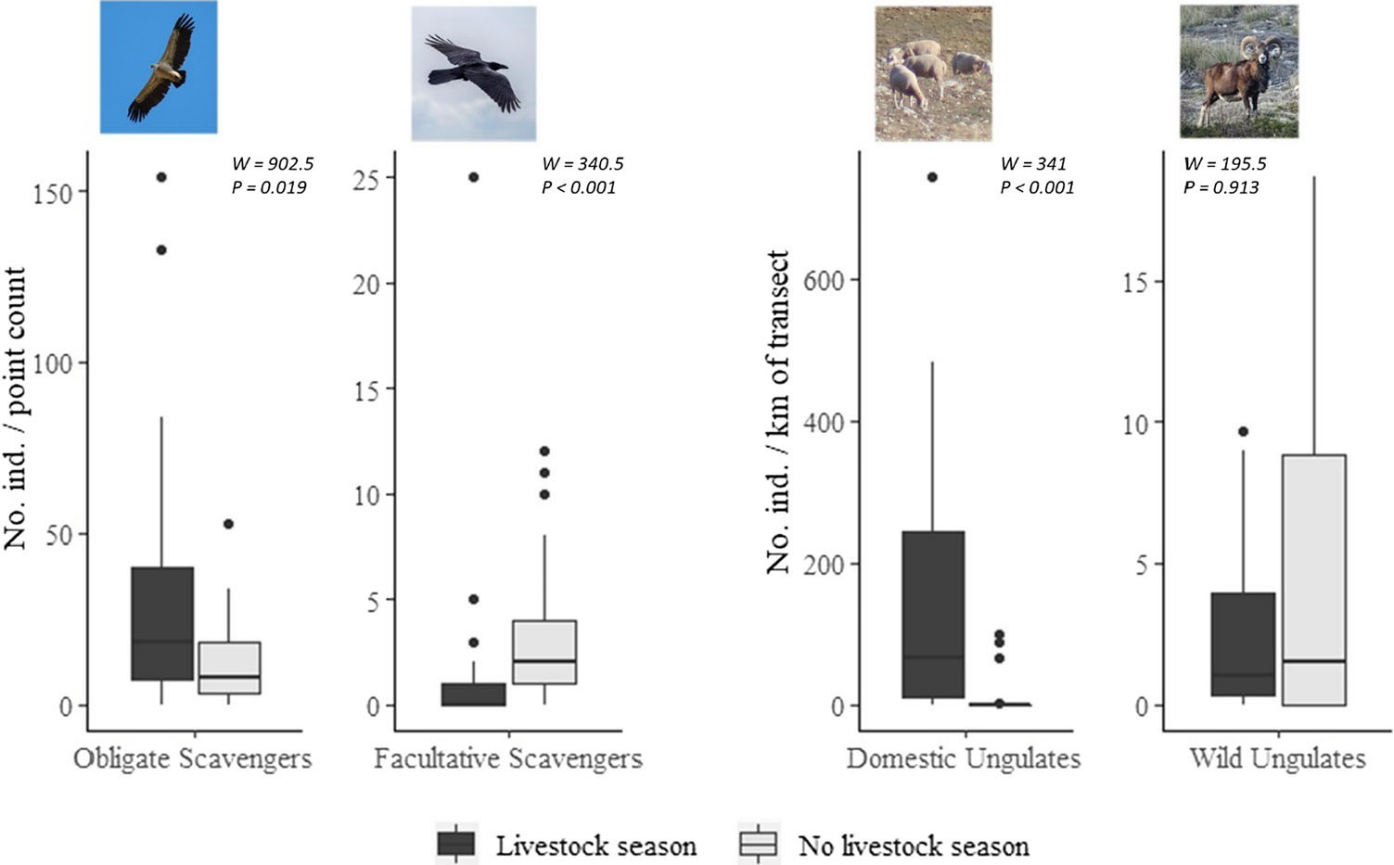
Abundance comparisons of (obligate and facultative) avian scavengers and (domestic and wild species) ungulates between seasons (livestock vs. no livestock) in the study area. Boxes include from the first to the third data quartiles; the median is represented by a horizontal line; thin lines extend from the hinge to the largest and smallest value no further than 1.5 * IQR (the interquartile range); points show outliers. Differences between seasons were calculated by the non-parametric Wilcoxon rank test (*α* = 0.05)

Obligate scavengers were more abundant during the livestock season (Wilcoxon rank test (*p* < 0.05); Fig. 2). We observed avian scavengers in 95.9% of the point counts, and totalled 1728 observed birds (range: 0–154 per point; mean ± SD: 23.4 ± 28.0; Table 1). We detected three species of obligate scavengers (griffon, black and bearded vultures) and five species of facultative scavengers. Griffon was by far the most abundant vulture (89.1% of all the observed birds), with 1106 individuals observed during the livestock season and 434 during the no livestock season (Table 1). In general, vultures were c. 2.5-fold more abundant during the livestock season than during the no livestock season (Fig. 2). In contrast, avian facultative scavengers were more abundant during the no livestock season (Fig. 2), with the carrion crow being the most frequently observed species, followed by common raven and golden eagle (Table 1).

**Table 1:** Results of the 30-min point counts conducted to survey avian scavengers’ abundance in the study area according to season (livestock and no livestock)

	Species	Livestock season				No livestock season			
		% Occurrence in points (n = 37)	Total individuals	Mean no. of ind./point	SD	% Occurrence in points (n = 38)	Total individuals	Mean no. of ind./point	SD
Obligate scavengers	Griffon vulture *Gyps fulvus*	91.89	1106	29.89	36.01	94.74	434	11.72	11.70
	Bearded vulture *Gypaetus barbatus*	10.81	9	0.24	0.89	15.79	6	0.16	0.37
	Cinereous vulture *Aegypius monachus*	2.70	1	0.03	0.16	2.63	1	0.03	0.16
Facultative scavengers	Golden eagle *Aquila chrysaetos*	0	0	0	–	23.68	10	0.27	0.51
	Common raven *Corvus corax*	16.22	8	0.22	0.53	23.68	16	0.43	0.90
	Carrion crow *Corvus corone*	35.14	41	1.11	3.78	63.16	93	2.51	3.18
	Eurasian jay *Garrulus glandarius*	2.70	1	0.03	0.16	0	0	0	−
	Eurasian magpie *Pica pica*	2.70	2	0.05	0.33	0	0	0	–
	Total	91.89	1168	31.57	36.00	100	560	15.14	12.41

Domestic ungulates were found in 55.0% of the transects, and totalled 10 046 animals (range: 0–2228 per transect; mean ± SD: 251.2 ± 479.3; Table 2). Sheep represented the bulk of the observed livestock species, followed far behind by goats. Cattle and horses were occasionally recorded. During the livestock season, domestic ungulates were c. 12-fold more abundant than during the no livestock season (Fig. 2). In contrast, wild ungulates were observed in more transects (70.0%), but total abundance (418 individuals; range: 0–56 per transect; mean ± SD: 10.45 ± 14.70) was much lower than that of domestic ungulates, and was similar between seasons (Fig. 2). Mouflons were the most abundant species during both seasons, followed by red and fallow deer, Iberian ibex and wild boar (Table 2).

**Table 2:** Results of the transects conducted to survey ungulates’ abundance in the study area according to season (livestock and no livestock). KAI: Kilometric Abundance Index

	Species	Livestock season			No livestock season		
		% Occurrence in transects (n = 20)	Total individuals	KAI	% Occurrence in transects (n = 20)	Total individuals	KAI
Domestic	Sheep *Ovis aries*	70	9038	150.63	15	760	12.67
	Goat *Capra aegagrus hircus*	35	194	2.73	5	1	0.02
	Cattle *Bos Taurus*	10	42	0.70	5	2	0.03
	Horse *Equus ferus caballus*	10	9	0.15	0	0	0
Wild	Mouflon *Ovis orientalis*	55	113	1.88	50	195	3.25
	Red deer *Cervus elaphus*	30	18	0.30	20	26	0.43
	Fallow deer *Dama dama*	10	13	0.22	20	37	0.62
	Iberian ibex *Capra pyrenaica*	10	2	0.03	5	1	0.02
	Wild boar *Sus scrofa*	5	13	0.22	0	0	0
	Total	100	9442	157.47	65	1022	17.03

The GLMM with higher performance included livestock season (Table 3), and supported the notion that griffon vulture abundance increased during the livestock season (Table 4). According to *R*^2^, season explained c. 33.0% of the variability in the number of observed griffon vultures on the plateau. Adding “nestling” period to the previous model did not improve R^2^ (Table 3), which suggests that the increased abundance of griffon vultures in the study area during the livestock season was not related to changes in breeder behaviour in relation to either the nesting season or movements of young vultures.

**Table 3:** Generalized linear mixed models (GLMMs) obtained from the AIC-based model selection to assess the factors influencing the changes in: (a) the number of griffon vultures observed in the study area per point count (“abundance”); (b) the GPS fixes per griffon vulture individual that fell within vs. beyond the study area limits (“GPS fixes”; see Methods for details on the explanatory variables)

Response variable	Model	k	AICc	delta-AIC	R^2^
Abundance	**season + (1|point)**	**1**	**1334.2**	**0**	**0.330**
	**season + nestling + (1|point)**	**2**	**1334.2**	**0.02**	**0.328**
	nestling + (1|point)	1	1507.2	172.97	
	(1|point)	0	1679.6	345.40	
GPS fixes	**breeding + season + year + (1|ind.)**	**5**	**8468.5**	**0**	**0.014**
	**breeding + season + sex + year + (1|ind.)**	**6**	**8468.7**	**0.17**	**0.040**
	season + year + (1|ind.)	4	8498.5	30.00	
	season + sex + year + (1|ind.)	5	8499.1	30.55	
	breeding + year + (1|ind.)	4	8735.2	266.70	
	breeding + sex + year + (1|ind.)	5	8735.3	266.79	
	year + (1|ind.)	3	8764.8	296.25	
	sex + year + (1|ind.)	4	8765.3	296.73	
	season + (1|ind.)	1	8795.4	326.89	
	season + sex + (1|ind.)	2	8796.0	327.48	
	breeding + season + (1|ind.)	2	8796.9	328.40	
	breeding + season + sex + (1|ind.)	3	8797.5	328.95	
	(1|ind.)	0	9099.5	631.00	
	sex + (1|ind.)	1	9100.1	631.51	
	breeding + (1|ind.)	1	9101.5	632.99	
	breeding + sex + (1|ind.)	2	9102.0	633.51	

Number of estimated parameters (k), AIC values, AIC differences (delta-AIC) with the highest ranked model (that with the lowest AIC) and the variability of the response variable explained by the predictors (R^2^) are shown

Bold: the selected models

**Table 4:** Selected generalized linear mixed models (GLMMs) showing the relation among season, nestling, breeding status and sex of griffon vultures, and year (see the text for details on the explanatory variables) and changes in: (a) the number of griffon vultures observed in the study area per point count (“abundance”); (b) the GPS fixes per griffon vulture individual that fell within vs. beyond the study area limits (“GPS fixes”)

Response variable	Model	Parameter	Estimate	SE	df
Abundance	Season + (1|point)	Intercept	2.188	0.161	3
		Season (livestock)	1.017	0.057	
	Season + nestling + (1|point)	Intercept	2.194	0.161	4
		Season (livestock)	0.955	0.071	
		Nestling (yes)	0.097	0.065	
GPS fixes	Breeding + season + year + (1|ind.)	Intercept	− 5.161	0.357	7
		Breeding (yes)	− 0.325	0.057	
		Season (livestock)	0.412	0.026	
		Year (2016)	0.317	0.027	
		Year (2017)	0.357	0.028	
		Year (2018)	− 0.193	0.040	
	Breeding + season + sex + year + (1|ind.)	Intercept	− 4.543	0.565	8
		Breeding (yes)	− 0.327	0.057	
		Season (livestock)	0.412	0.026	
		Sex (male)	− 0.981	0.712	
		Year (2016)	0.317	0.027	
		Year (2017)	0.357	0.028	
		Year (2018)	− 0.193	0.040	

Only the models with the highest R^2^ are shown. The estimate of the parameters (including the sign), the standard error of the parameters (SE) and the degrees of freedom of the models (df) are shown

### Seasonal changes in the foraging areas of the GPS-tracked griffon vultures

**Fig. 3:**
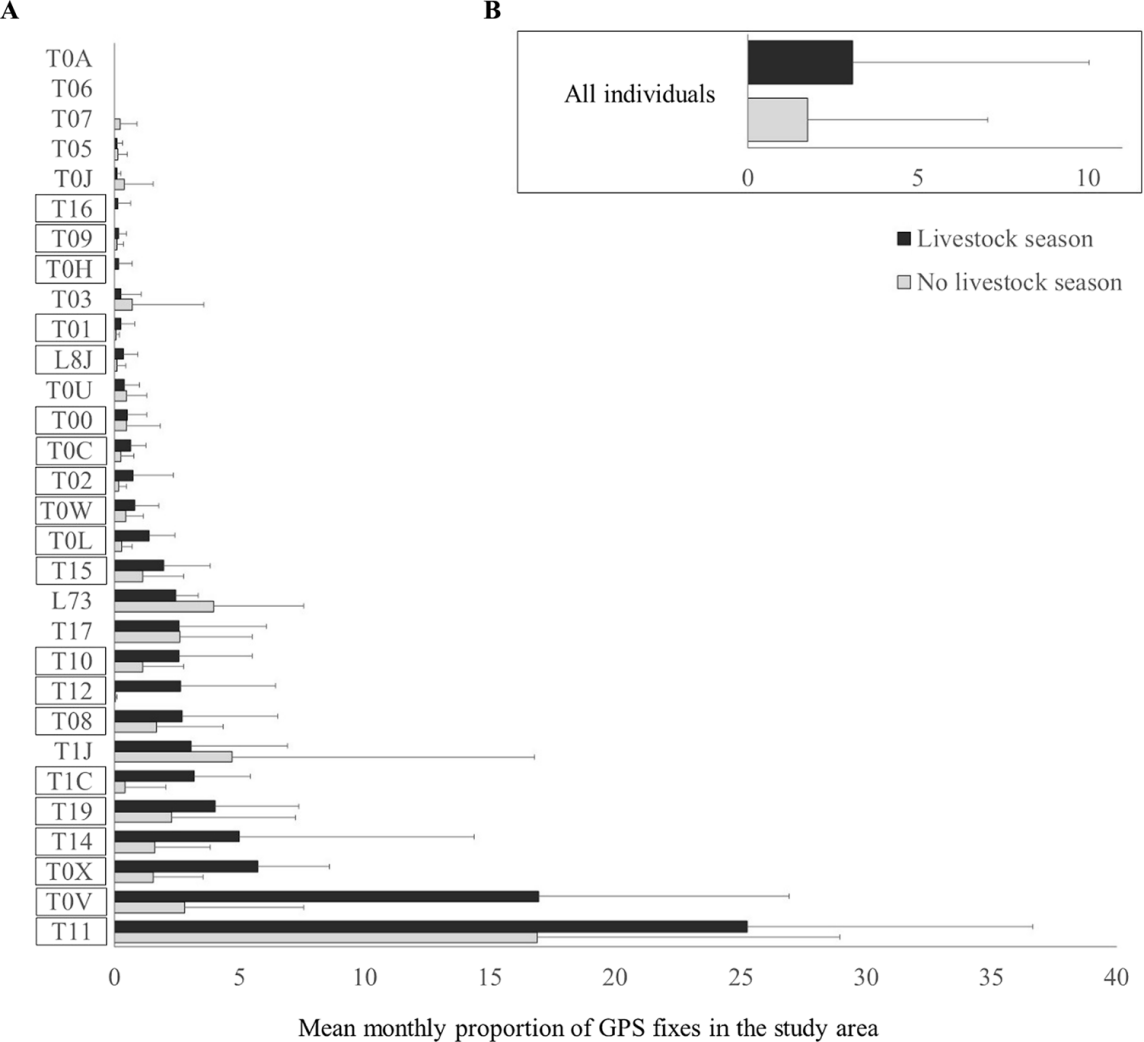
GPS fixes of the tracked griffon vultures in the study area during the livestock and no livestock seasons per tracked individual (**A**) and for all the tracked individuals together (**B**). Bars show the average of the monthly proportion of GPS fixes in relation to the total fixes; thin lines show the standard error. The individuals for which the proportion of fixes in the study area is higher during the livestock season than during the no livestock season are highlighted with boxes

Griffon vultures tracking generated 11 805 GPS fixes in the study area and 544 429 outside it. According to the GLMM with higher performance, the proportion of inside vs. outside fixes depended on vulture breeding status, livestock season, sex, and year, as well as individual as random factor (Table 3). In particular, the proportion of GPS fixes inside the study area increased during the livestock season (Fig. 3), especially for non-breeding vultures and females, with some variation among years (Table 4). However according to R^2^, this model had low explanatory capacity (4%), which became lower after excluding the variable “sex” (Table 4). During the livestock season, the proportion of GPS fixes inside vs. outside the study area increased for 19 individuals (c. 63% of the GPS-tracked individuals; Fig. 3A).

## Discussion

The results obtained from field observations and the GPS-tracked griffon vultures revealed that vulture (mostly griffons) abundance in the studied pastureland increased after transhumant herds had arrived. This agrees with previous research conducted in northern Spain, which found that the occurrence of griffon vultures increased at the roosting sites near transhumant sheep’s summer pastures (Olea and Mateo-Tomás 2009). In contrast, the presence of transhumant livestock did not lead to an increase in the abundance of observed avian facultative scavengers. This was true even for species that frequently scavenge, such as the golden eagle, which is a regular carrion consumer in nearby Mediterranean mountains where griffon vultures are absent (Blázquez et al. 2009; Sánchez-Zapata et al. 2010). In the study area, however, golden eagles do not feed on livestock carcasses, likely because vultures quickly consume them (Arrondo et al. 2019). The high abundance of vultures here, especially during the livestock season, could therefore prevent other less efficient scavengers from responding to transhumant herds, as found in other areas and with other carcass types (Sebastián-González et al. 2013; Morales-Reyes et al. 2017).

These seasonal differences in carrion availability were probably perceived by griffon vultures, as c. two thirds of the GPS-tracked individuals increased their use of the study area during the livestock season (Fig. 3). However, our models indicated high inter-individual variability, so there must be other variables associated with the tracked individuals that influenced their movement, such as carrion availability outside the study area. In fact, this area is very small (c. 150 km^2^) compared to the griffon vulture home range in southern Spain (average = c. 11 000 km^2^; Arrondo et al. 2020b). Though this small area congregates more than 35 000 transhumant ungulates every summer, vultures may also find abundant food in many other areas within their home ranges (Arrondo et al. 2018, 2020b). For instance, Martin-Díaz et al. (2020) found that griffon vultures from south-eastern Spain widely forage in big game areas, feeding on wild ungulate carcasses from hunting activities. However, the main hunting season, which takes place in autumn–winter, overlaps only partially with the presence of transhumant herds in our study area. Outside the hunting season, griffon vultures greatly tend to forage in grasslands with abundant livestock (Martin-Díaz et al. 2020), as it is the case of our study area. Regardless, griffon vultures’ observed individual response to transhumant livestock sufficed to trigger the above-mentioned strong seasonal variation in vulture abundance in the study area at the landscape level. An alternative, non-mutually exclusive explanation is that griffon vultures from other populations could also be attracted by transhumant herds to our study area, thus contributing to the increased vulture numbers there during the livestock season.

We also found that non-breeding and female griffon vultures were the individuals that responded the most to the presence of transhumant herds. On one hand, most of the griffon vultures observed using roosts near transhumant livestock in northern Spain were immature (Olea and Mateo-Tomás 2009). However, why breeding vultures respond less to an abundant food resource that is very close to their nesting sites is difficult to explain. On the other hand, griffon vultures have very slight sexual dimorphism (Xirouchakis and Poulakakis 2008) and share chick-rearing investment (Xirouchakis and Mylonas 2007). Thus, finding differences between sexes was an unexpected result. This contrasts to other vulture species with marked sexual dimorphism, such as Andean condors (*Vultur gryphus*), which show different resource exploitation patterns (Perrig et al. 2021). However, recent studies suggest that sex-partitioning in resource exploitation on a fine scale should not be ruled out in griffons (Arrondo et al. 2020a, b). In any case, our results do not support a strong influence of the studied individual traits on griffon vulture responses to transhumant livestock.

Transhumance implies that the presence of domestic ungulates is drastically different between seasons. Although the seasonal absence of livestock could facilitate access of wild ungulates to the plateau after release from pasture competition, which occurs in other areas (Chirichella et al. 2014), our results showed that wild ungulates’ seasonal abundance did not change seasonally. This might be due to the harsh climatic conditions in winter, which limits pasture availability in the plateau. In autumn–winter, some big game hunting takes place in the study area, which could make wild ungulates more cautious during this period. This generally means that when transhumant livestock leaves the plateau, carrion availability in the study area sharply declines, which leads to vultures’ lower abundance and foraging activity.

Overall, our findings indicate that pasturelands with extensive livestock are important foraging areas for vultures. Although livestock farming has historically been involved in landscape transformation, extensive livestock farming might support high biodiversity values compared to intensive farming practices (Bernués et al. 2016). In particular, extensive farming modulates plant diversity in mountain pastures (Komac et al. 2014) and limits the vegetation succession in rewilding contexts (Riedel et al. 2013). Nevertheless, the global trend of increasing resources demand to supply the growing human population drives the intensification of production systems. Thus, the risk of traditional livestock practices, such as transhumance, being completely replaced with more intensive techniques in the near future is high, especially in developed countries. This may seriously compromise the sustainable development of human societies, as intensive farming demands high inputs of energy, water, and feed, which is linked to land degradation, water pollution, greenhouse gas emission, and, eventually, biodiversity loss (Ilea 2009). Thus, if the rewilding process is not properly managed, this situation could compromise not only vultures’ conservation, but also the maintenance of other values associated with traditional activities, including local ecological knowledge (Oteros-Rozas et al. 2013b). In Mediterranean areas, the social stakeholders linked to extensive farming systems (e.g. shepherds) positively value scavengers’ role (mostly vultures) as providers of ecosystem services (Cortés-Avizanda et al. 2018; Morales-Reyes et al. 2018). Therefore, loss of traditional practices as a result of disconnecting people from nature could imply a negative impact on the perception of the scavenger guild (Gigante et al. 2021) and a rise in harmful practices for scavengers, such as poisoning (Brink et al. 2021). Moreover, with the decline of extensive farming systems and the reduction of grassland areas in abandoned rural areas, scavenging birds will be driven to exploit resources in more anthropized landscapes, where the physiological condition and survival rates of vultures are lower (Arrondo et al. 2020b; Gangoso et al. 2021).

Until rewilding processes may lead to natural landscapes where wild ungulate carrion is made abundantly accessible to avian scavengers, it is essential for scavenger conservation to maintain extensive livestock farming against further development of intensive farming practices. This requires addressing the causes that are driving traditional farming systems towards extinction. Among them, the low profitability of the extensive livestock’s product stands out as the main concern for Spanish farmers (Oteros-Rozas et al. 2013a). Hence, agro-economic policies need to be aimed at enhancing the market value of products by creating quality seals and effective publicity campaigns. In our study area, there is a system of public incentives, from both the European Union and regional administrations, that contributes to support transhumance, as well as an educational centre specialized in training young shepherds. These strategies have successfully helped to maintain a relatively important transhumant activity in this area, so they could also be implemented elsewhere. At the same time, intensive farming should be subject to stricter regulatory policies that allow minimize their multiple negative impacts on ecosystems and rural communities (Leip et al. 2015). Consequently, a more comprehensive understanding of the ecological consequences of abandoning traditional farming practices is crucial for environmental managers and policy makers to harmonize human activities and biodiversity in the current global ecological transition.

## Supplementary Information

Below is the link to the electronic supplementary material.

Supplementary file1 (PDF 128 kb)
