# Supplementary material for: The value of transhumance for biodiversity conservation: Vulture foraging in relation to livestock movements
Source: Ambio. 2021 Dec 7;51(5):1330–42. doi: 10.1007/s13280-021-01668-x (PMC8931130; doi:10.1007/s13280-021-01668-x)
Supplement: Supplementary file 1 — Supplementary file1 (PDF 128 kb) [file 13280_2021_1668_MOESM1_ESM.pdf]

Supplementary material for

The value of transhumance for biodiversity conservation: vulture foraging in relation to livestock movements

Index

Table S1. GPS device settings and time elapsed between consecutive locations.

Table S2. Details of griffon vultures tagged and tracking.

Table S1. GPS device settings and time elapsed between consecutive locations. Devices were activated one hour before sunrise and turned off one hour after sunset. High-performance settings were active continuously except if we detected a low battery status for several days. We then activated the low-performance setting until the battery recovered normal status. During the study period, the time between consecutive locations ranged from 5 seconds to 14.9 h with a median of 5 min.

	Battery status			
	Full	Non-full	Close to security level	Under security level
Low-performance setting	10 min	30 min	1 h	1 day
High-performance setting	5 min	20 min	30 min	1 day

Table S2. Details of griffon vultures tagged and tracking. The table shows fixes recorded during the period 2015-2018, the number of monitored months and the number of breeding years for each tagged individual.

Individual	Sex	No. fixes	No. months	No. breeding years
L73	Male	35539	49	4
L8J	Male	5552	45	1
T00	Female	11043	49	4
T01	Female	10185	45	3
T02	Male	14850	22	2
T03	Male	6764	40	3
T05	Male	49161	49	4
T06	Male	8965	9	0
T07	Male	3177	14	1
T08	Female	22062	47	4
T09	Male	42442	49	4
T0A	Male	20769	46	3
T0C	Male	56490	49	3
T0H	Male	31780	49	1
T0J	Female	37920	49	3
T0L	Male	11991	9	0
T0U	Female	18603	25	2
T0V	Male	5551	43	3
T0W	Female	9778	14	2
T0X	Male	15831	22	2
T10	Female	6030	11	1
T11	Male	21468	49	0
T12	Male	8197	33	3
T14	Male	6747	43	1
T15	Female	36000	49	3
T16	Male	779	33	1
T17	Male	22698	49	0
T19	Female	9471	23	2
T1C	Female	5751	32	3
T1J	Female	20640	49	3
